# Complete Chloroplast Genome of *Tanaecium tetragonolobum*: The First Bignoniaceae Plastome

**DOI:** 10.1371/journal.pone.0129930

**Published:** 2015-06-23

**Authors:** Alison Gonçalves Nazareno, Monica Carlsen, Lúcia Garcez Lohmann

**Affiliations:** 1 Universidade de São Paulo, Instituto de Biociências, Departamento de Botânica, São Paulo, São Paulo, Brazil; 2 University of Missouri-St. Louis, Biology Department, St. Louis, Missouri, United States of America; ICGEB, INDIA

## Abstract

Bignoniaceae is a Pantropical plant family that is especially abundant in the Neotropics. Members of the Bignoniaceae are diverse in many ecosystems and represent key components of the Tropical flora. Despite the ecological importance of the Bignoniaceae and all the efforts to reconstruct the phylogeny of this group, whole chloroplast genome information has not yet been reported for any members of the family. Here, we report the complete chloroplast genome sequence of *Tanaecium tetragonolobum* (Jacq.) L.G. Lohmann, which was reconstructed using *de novo* and referenced-based assembly of single-end reads generated by shotgun sequencing of total genomic DNA in an Illumina platform. The gene order and organization of the chloroplast genome of *T*. *tetragonolobum* exhibits the general structure of flowering plants, and is similar to other Lamiales chloroplast genomes. The chloroplast genome of *T*. *tetragonolobum* is a circular molecule of 153,776 base pairs (bp) with a quadripartite structure containing two single copy regions, a large single copy region (LSC, 84,612 bp) and a small single copy region (SSC, 17,586 bp) separated by inverted repeat regions (IRs, 25,789 bp). In addition, the chloroplast genome of *T*. *tetragonolobum* has 38.3% GC content and includes 121 genes, of which 86 are protein-coding, 31 are transfer RNA, and four are ribosomal RNA. The chloroplast genome of *T*. *tetragonolobum* presents a total of 47 tandem repeats and 347 simple sequence repeats (SSRs) with mononucleotides being the most common and di-, tri-, tetra-, and hexanucleotides occurring with less frequency. The results obtained here were compared to other chloroplast genomes of Lamiales available to date, providing new insight into the evolution of chloroplast genomes within Lamiales. Overall, the evolutionary rates of genes in Lamiales are lineage-, locus-, and region-specific, indicating that the evolutionary pattern of nucleotide substitution in chloroplast genomes of flowering plants is complex. The discovery of tandem repeats within *T*. *tetragonolobum* and the presence of divergent regions between chloroplast genomes of Lamiales provides the basis for the development of markers at various taxonomic levels. The newly developed markers have the potential to greatly improve the resolution of molecular phylogenies.

## Introduction

Chloroplasts carry out photosynthesis, representing one of the most essential organelles in green plants and algae [1]. This plastid contains a circular double-stranded DNA molecule of 115 to 165 kb in length [2]. Chloroplast genomes typically present a conserved quadripartite structure composed of a large single copy (LSC) region and a small single copy (SSC) region, which are separated by two copies of inverted repeats (IRs) [3]. Causes of variation in chloroplast genome size include gene and intron gains and losses [4,5], expansion/contraction of the IR [6–11], and major structural rearrangements such as inversions [3,12,13] and transpositions [14]. Nonetheless, the gene content of plastid genomes may be similar even between distantly related species. Chloroplast genomes generally contain 110 to 130 genes encoding up to 80 unique proteins, four ribosomal RNAs, and approximately 30 transfer RNAs [3,15].

Since the first publication of the chloroplast genomes of tobacco (*Nicotiana tabacum*, [16]) and the umbrella liverwort (*Marchantia polymorpha*, [17]), more than 530 complete plastid genomes, from a wide diversity of taxonomic groups were sequenced (Organelle Genome Resources Database, http://ncbi.nlm.nih.gov/genome/organelle/). Although the number of complete chloroplast genomes sequenced has almost doubled in the last years (Organelle Genome Resources Database, http://ncbi.nlm.nih.gov/genome/organelle/), especially due to progress in DNA sequencing technologies, only a small fraction of botanical families have had their whole chloroplast genomes sequenced and carefully described. Indeed, almost half of all plastid genomes deposited at GenBank belong to only nine plant families (i.e., Asteraceae, Brassicaceae, Fabaceae, Magnoliaceae, Malvaceae, Myrtaceae, Pinaceae, Poaceae, and Theaceae). On the other hand, many more plant families, especially key tropical groups (e.g., Bignoniaceae, Bromeliaceae, Lauraceae, and Lecythidaceae) remain unrepresented in GeneBank. In view of the advancements in high-throughput next-generation DNA sequencing technologies [18,19], and the ability to accurately assemble new chloroplast genomes for non-model organisms, the number of whole plastid genomes will probably rise exponentially in coming years, decreasing the sampling gaps currently seen in Global databases.

The Bignoniaceae is predominantly tropical, and includes approximately 80 genera and 840 species of trees, shrubs, vines and woody lianas [20]. It belongs to the order Lamiales, which includes ca. 24,000 species [21]. Even though 27 chloroplast genomes are available for other families within the order (e.g., Gesneriaceae, Oleaceae, and Pedaliaceae), not a single chloroplast genome has been fully sequenced for a member of the Bignoniaceae. Among the eight major clades that are currently recognized within the family [22], the tribe Bignonieae *sensu stricto* is the largest and most important ecologically, accounting for 393 species and 21 genera [23]. Bignonieae is one of the largest clades of neotropical lianas, representing an ideal model for evolutionary studies due to their wide distribution and high levels of ecological and morphological diversity [24]. A broad scale study of phylogenetic relationships within Bignonieae using chloroplast (*ndhF*) and nuclear (*PepC*) DNA sequences [24] has provided important insights into the systematics [23], biogeography [25], community structure [26], evolution of development [27], and morphological evolution [28–30] within this group, phylogenetic resolution within most of its genera remains unclear. Whole chloroplast genome sequences for members of the tribe Bignonieae provide key information for finer-scale relationships within this tribe and broader-scale studies in the whole Bignoniaceae. Complete chloroplast genome sequences have been broadly used for phylogenetic studies in the Poaceae [31,32], and Asteraceae [8].

By using next-generation sequencing technology and applying a combination of *de novo* and reference-guided assembly, we were able to reconstruct the whole genome sequence for *Tanaecium tetragonolobum* (Bignonieae, Bignoniaceae). *Tanaecium tetragonolobum* is an insect-pollinated and water-dispersed species of liana [23]. It belongs to a genus that includes 17 species [23], ten of which have been sampled in the current molecular phylogeny of the tribe [24]. Members of *Tanaecium* have variable distribution patterns, ranging from Central America to the northern half of South America [23, 33]. Details of its chloroplast genome structure and organization are reported and compared with previously annotated Lamiales plastomes. *Tanaecium tetragonolobum* is the focal taxon of a detailed phylogeographic study in the Amazon and the findings of the present study will help other areas of molecular systematics.

## Material and Methods

### Collecting material and DNA sequencing

The specimen *Lohmann 619* of *Tanaecium tetragonolobum* was collected with a collecting permit from the “Instituto Nacional de Recursos Naturales” (INRENA); duplicates of this material are deposited at MO and MOL. As this study does not involve a threatened plant species, no additional permits from regulatory authorities from Peru concerned with the protection of threatened wildlife were required. Total genomic DNA was extracted using a mini-scale CTAB protocol [34]. 5 μg of total DNA were fragmented using a Covaris S-series sonicator, and short-insert (300 bp) libraries were constructed with NEBNext DNA Library Prep Master Mix Set and NEBNext Multiplex oligos for Illumina (New England BioLabs Inc., Ipswich, MA) following the manufacturer’s protocol. To verify the expected size profile, library products were run against a size standard on a 1% low-melt agarose gel at 120 V for 30 min. DNA library concentration was determined using the Kapa Library Quantification Kit (Kapa Biosystems Inc., Wilmington, MA) on an Applied Biosystems 7500 Real-Time PCR System. The library of *T*. *tetragonolobum* was diluted to a concentration of 10 nM, pooled together with other 19 non-target species in one lane, and sequenced (single end) on an Illumina HiSeq 2000 system (Illumina Inc., San Diego, CA) at the University of São Paulo (Escola Superior de Agricultura Luiz de Queiroz da Universidade de São Paulo) in Piracicaba, Brazil.

### Genome assembly and annotation

Illumina adaptors and barcodes were removed from raw reads. The clean reads were then filtered for quality using a custom Perl script that trimmed reads from the ends until there were three consecutive bases with a Phred quality score >20. Reads with a median quality score of 21 or less, with more than three uncalled bases, or less than 40 bp in length were removed from the dataset. The chloroplast genome of *T*. *tetragonolobum* was reconstructed using a combination of *de novo* and reference-guided assemblies. Clean and high-quality sequence reads were assembled *de novo* using Velvet 2.3 [35], with a *K*-mer length value of 71. A reference-guided assembly was performed using YASRA 2.32 [36] using *Olea europaea* L. (Oleaceae, Lamiales, GenBank accession number NC_013707) as reference. Contigs produced *de novo* were blasted against the original chloroplast genome reference in order to exclude contigs of nuclear origin. Contigs with coverage below 10x were eliminated, likely leading to the exclusion of contigs of mitochondrial origin as well. The remaining *de novo* and reference-guided contigs were assembled into larger contigs in Sequencher 5.3.2 (Gene Codes Inc., Ann Arbor, MI) based on at least 20 bps overlap and 98% similarity. Any discrepancies between *de novo* and reference-guided contigs were corrected by searching the high quality read pool using the UNIX ‘grep’ function. The ‘grep’ function was also used to find reads that could fill any gaps between contigs that did not assemble in the initial set of analyses (i.e., genome walking technique). We then applied Jellyfish [37] to create a 20-kmer count look-up table that was used as basis to check for the quality of the *T*. *tetragonolobum* chloroplast genome sequences. Genome coverage was also analyzed using Jellyfish, which resulted in a 127-fold genome coverage.

The chloroplast genome of *T*. *tetragonolobum* was annotated using DOGMA (Dual Organellar GenoMe Annotator, http://dogma.ccbb.utexas.edu/, [38]), with manual corrections for potential changes in the start and stop codons, as well as intron positions based on comparisons to homologous genes in other plastomes. Transfer RNA genes were identified with DOGMA [38] and the tRNAscan-SE program ver. 1.23 (http://lowelab.ucsc.edu/tRNAscan-SE/, [39]). We used CpBase (http://chloroplast.ocean.washington.edu/) to determine the functional classification of the chloroplast genes. A circular representation of the *T*. *tetragonolobum* chloroplast genome was made using GenomeVx tool (http://wolfe.ucd.ie/GenomeVx/, [40]). The whole nucleotide sequence of the *T*. *tetragonolobum* plastome along with gene annotations was deposited in GenBank (accession number KR534325). The short read library of *T*. *tetragonolobum* is available from the ENA read archive under accession number ERS717260.

### Comparative analyses with other Lamiales chloroplast genomes

The software mVISTA (http://genome.lbl.gov/vista/mvista/submit.shtml, [41]) was used in Shuffle-LAGAN mode [41] to compare the complete cp genome of *T*. *tetragonolobum* with three representatives of chloropast genomes of other species of Lamiales: *Boea hygrometrica* (Bunge) R. Br. (Gesneriaceae; NC_016468), *Olea europaea* (Oleaceae; NC_013707), and *Sesamum indicum* L. (Pedaliaceae; NC_016433). The closely related but basal species *Nicotiana tabacum* L. (Solanaceae; Solanales; NC_001879) was used as reference in the comparative analyses.

In order to examine variation in the evolutionary rates of chloroplast genes, we calculated the non-synonymous substitution rates (Ka), synonymous substitution rates (Ks), and their ratio (Ka/Ks) using Model Averaging in the KaKs_Calculator program [42]. Protein-coding sequences from *T*. *tetragonolobum* and three Lamiales species (*B*. *hygrometrica*, *O*. *europaea*, and *S*. *indicum*) were aligned using the software MAFFT v.7 [43]. The corresponding genes of *N*. *tabacum* were used as reference in the alignments.

### The repeat structure of the chloroplast genome of *Tanaecium tetragonolobum* and microsatellite primer design

We used the online REPuter software (http://bibiserv.techfak.uni-bielefeld.de/reputer, [44]) to identify and locate forward, palindrome, reverse, and complement sequences with n ≥30 bp and a sequence identity ≥90%. To assess the number of repeats in other chloroplast genomes, we ran the same REPuter analyses against the chloroplast genomes of the other three Lamiales species that were used in the comparative analyses. Simple sequence repeats (SSRs) were identified using the online software WebSat (http://wsmartins.net/websat/, [45]) and Gramene Ssrtool (http://archive.gramene.org/db/markers/ssrtool, [46]). We applied a threshold seven to mononucleotide repeats, four to dinucleotide repeats and three to, tri-, tetra-, penta-, and hexanucleotide repeats. Additionally, a potential set of microsatellite markers was identified for *T*. *tetragonolobum*. Primers were designed with the software PRIMER3 (http://bioinfo.ut.ee/primer3-0.4.0/, [47]) by setting product size ranges from 100 to 250 bp, primer size from 18 to 24 bp, GC content from 40 to 60, and 1°C as the maximum difference between the melting temperatures of the left and right primers. To identify variation in the set of chloroplast SSRs markers designed for *T*. *tetragonolobum*, we searched for the same loci in the cp genomes of *Boea hygrometrica*, *Olea europaea*, and *Sesamum indicum*.

## Results and Discussion

### Genome content and organization

**Fig 1:**
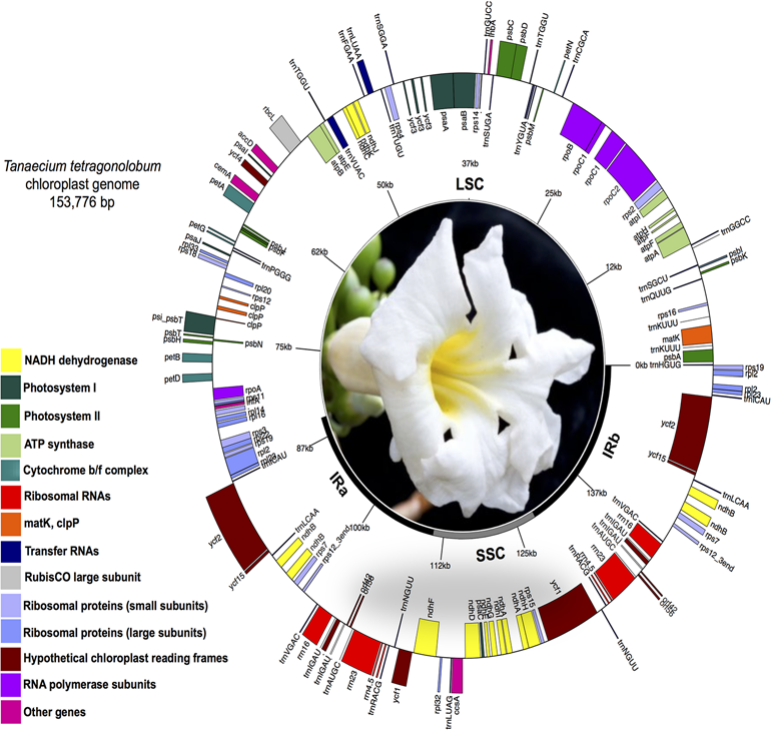
Circular map of the chloroplast genome of *Tanaecium tetragonolobum* (Jacq.) L.G. Lohmann. Genes drawn within the circle are transcribed clockwise, while genes drawn outside are transcribed counterclockwise. Genes belonging to different functional groups are color-coded. Dark bold lines indicate inverted repeats (IRa and IRb) that separate the genome into large (LSC) and small (SSC, bold grey line) single copy regions. Drawn using GenomeVx (Conant and Wolfe 2008).

The size of the chloroplast genome of *T*. *tetragonolobum* is 153,776 bp with a typical quadripartite structure, including a LSC region of 84,612 bp and a SSC region of 17,586 bp separated by a pair of identical IRs of 25,789 bp each (Fig 1). This chloroplast genome size is consistent with those from other flowering plants, which range from 125,373 bp in *Cuscuta exaltata* [4] to 176,045 bp in *Vaccinium macrocarpon* [48]. The GC content of the chloroplast genome of *T*. *tetragonolobum* is 38.3%, although this value is slightly higher in IR regions (43.0%) and lower in the LSC (36.5%) and SSC regions (33.1%). The CG content of *T*. *tetragonolobum* is the highest content among the Lamiales species studied here (Table 1) but slightly lower than other angiosperms, such as *Paeonia obovata* (38.43%; [7]).

names represent SSR loci shared with *Sesamum indicum*.

**Table 1:** Comparison of chloroplast genomes among four species of Lamiales.

	*Tanaecium tetragonolobum* (Bignoniaceae)	*Boea hygrometrica* (Gesneriaceae)	*Olea europaea* (Oleaceae)	*Sesamum indicum* (Pedaliaceae)
Characteristics				
Size (base pair; bp)	153,776	153,493	155,889	153,338
LSC length (bp)	84,612	84,692	86,614	85,180
SSC length (bp)	17,586	17,901	17,791	17,874
IR length (bp)	25,789	25,450	25,742	25,142
GC content (%)	38.3	37.6	37.8	38.2
Number of genes	121	131	122	122
Protein-coding genes	85	94	86	86
Structure RNAs	35	37	36	36
Genes with intron(s)	13	19	18	18
Coding rRNAs genes (%bp)	5.85	5.89	5.80	5.89
Coding tRNAs genes (%bp)	1.81	1.88	1.88	1.78
Coding protein genes (%bp)	51.21	52.98	52.58	52.68
Noncoding regions (%bp)	41.13	39.25	39.74	39.65
References	This study	[79]	[47]	[10]

**Fig 2:**
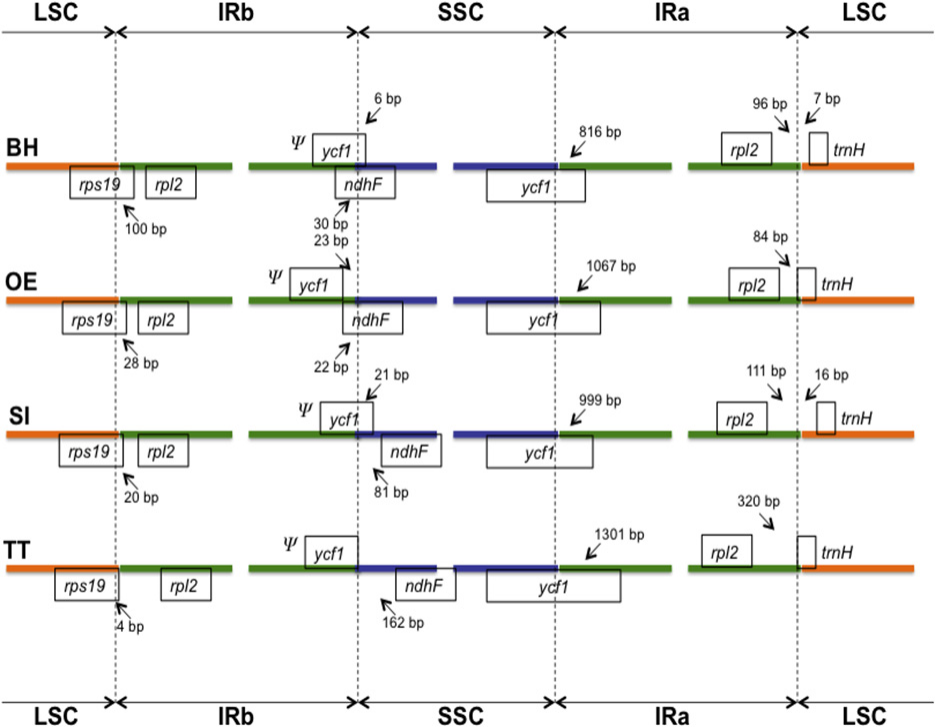
Comparison of boundary positions between single copy (large, LSC or small, SSC) and inverted repeat (IR) regions among four Lamiales genomes. Genes above lines are transcribed forward while genes below the lines are transcribed reversely. BH: *Boea hygrometrica*; OE: *Olea europaea*; SI: *Sesamum indicum*, and TT: *Tanaecium tetragonolobum*. *Ψ* indicates a pseudogene.

The chloroplast genome of *T*. *tetragonolobum* contains 121 genes in total (Table 2). Eighty-six of them are unique protein-coding genes, representing 79,020 nucleotides coding for 26,340 codons. Ten of these protein-coding genes are located within the IR region, and thus fully duplicated within the genome, including *rpl2*, *rpl23*, *ycf2*, *ycf15*, *ndhB*, *rps7*, *rps12_3end*, *ycf68*, *orf42*, and *orf56*. Additionally, 31 unique transfer RNA genes (tRNAs), representing all 20 aminoacids are distributed throughout the genome; one in the SSC region, 23 in the LSC region and seven in the IR region. Four ribosomal RNA genes (rRNAs) were also identified in this genome, all of them located in the IR regions. Sequence analyses indicated that 51.21% of the genome sequences encode for proteins, 1.81% for tRNAs, and 5.85% for rRNAs, whereas the remaining 41.13% are noncoding, representing introns, intergenic spacers and pseudogenes such as *ycf1*. Among all genes, eleven have a single intron (seven protein-coding and four tRNA genes) and two protein-coding genes (*clpP* and *ycf3*) have two or more introns. Out of the genes with introns, seven are located in the LSC region (five protein-coding and two tRNAs), four in the SSC region (two protein-coding and two tRNAs), and one protein-coding gene (*ndhA*) in the IR region. The *rps12* gene is trans-spliced, with the 5’ end located in the LSC and the 3’ end duplicated in the IR regions; this same pattern was also reported in other plant species, including *Olea europaea* [49]. Among all genes, the *trnK-UUU* has the largest intron (2,490 bp), which contains the protein-coding gene *matK*. Similar to other flowering plants [7,10,50], *T*. *tetragonolobum* has two genes (*rps19* and *trnH*) located in the position of IR/LSC junctions (Fig 2). This pattern is different in monocots, all of which usually have a fully duplicated *rps19* gene in the IR/LSC junctions [51]. We also observed eight cases of overlapping genes (*psbD/psbC*, *ndhK/ndhC*, *trnP-UGG/trnP-GGG*, *clpP/psi_psbT*, *rpoA/rps11*, *rps3/rpl2*, *rps12/rps12_3end*, *orf88/ndhA*).

**Table 2:** One hundred and twenty-one genes contained within the chloroplast genome of *Tanaecium tetragonolobum* (Jacq.) L.G. Lohmann.

Function	Gene group	Gene name			
**Self replication**	Ribosomal RNA genes	*rrn4*.*5*	*rrn5*	*rrn16*	*rrn23*
	Transfer RNA genes	*trnA-UGC* ^*^	*trnC-GCA*	*trnD-GUC*	*trnE-UUC*
		*trnF-GAA*	*trnG-GCC*	*trnG-UCC*	*trnH-GUG*
		*trnI-CAU*	*trnI-GAU* ^*^	*trnK-UUU* ^*^	*trnL-CAA*
		*trnL-UAA* ^*^	*trnL-UAG*	*trnfM-CAU*	*trnM-CAU*
		*trnN-GUU*	*trnP-UGG*	*trnP-GGG*	*trnQ-UUG*
		*trnR-ACG*	*trnR-UCU*	*trnS-GCU*	*trnS-GGA*
		*trnS-UGA*	*trnT-GGU*	*trnT-UGU*	*trnV-GAC*
		*trnV-UAC* ^*^	*trnW-CCA*	*trnY-GUA*	
	Small subunit of ribosome	*rps2*	*rps3*	*rps4*	*rps7*
		*rps8*	*rps11*	*rps12*	*rps14*
		*rps15*	*rps16* ^*^	*rps18*	*rps19*
		*rps12_3end*			
	Large subunit of ribosome	*rpl12* ^*^	*rpl14*	*rpl16*	*rpl20*
		*rpl22*	*rpl23*	*rpl32*	*rpl33*
		*rpl36*			
	RNA polymerase subunits	*rpoA*	*rpoB*	*rpoC1* ^*^	*rpoC2*
**Photosynthesis**	NADH dehydrogenase	*ndhA* ^*^	*ndhB* ^*^	*ndhC*	*ndhD*
		*ndhE*	*ndhF*	*ndhG*	*ndhH*
		*ndhI*	*ndhJ*	*ndhK*	
	Photosystem I	*psaA*	*psaB*	*psaC*	*psaI*
		*psaJ*	*ycf3* ^*^	*psi_psbT*	
	Photosystem II	*psbA*	*psbC*	*psbD*	*psbE*
		*psbF*	*psbH*	*psbI*	*psbJ*
		*psbK*	*psbL*	*psbM*	*psbN*
		*psbT*	*psbZ*		
	Cytochrome b/f complex	*petA*	*petB*	*petD*	*petG*
		*petL*	*petN*		
	ATP synthase	*atpA*	*atpB*	*atpE*	*atpF* ^*^
		*atpH*	*atpI*		
	Large subunit of rubisco	*rbcL*			
**Other genes**	Translational initiation factor	*infA*			
	Maturase	*matK*			
	Protease	*clpP* ^*^			
	Envelope membrane protein	*cemA*			
	Subunit of acetyl-CoA-carboxylase	*accD*			
	c-type cytochrome synthesis	*ccsA*			
	Component of TIC complex	*ycf1* ^*^			
**Unknown function**	Hypothetical chloroplast reading frames	*ycf2*	*ycf4*	*ycf15*	*ycf68*
	ORFs	*orf42*	*orf56*	*orf188*	
	Other(s)	*ihbA*			

Asterisks indicate genes containing one or more introns.

### Comparison with other Lamiales chloroplast genomes

The availability of three other complete chloroplast genomes of Lamiales (*Boea hygrometrica*, *Olea europaea*, and *Sesamum indicum*) provided an opportunity to compare the chloroplast genome organization and sequence variation within the order. The chloroplast genome was rather conserved within Lamiales, and neither inversions nor translocations were detected in the four genomes analyzed. Similar to other flowering plants, the IR region was more conserved in these species than the LSC and SSC regions. In addition, we also observed some differences within Lamiales in terms of genome size, gene and intron losses, and IR expansion and contraction. In terms of genome size, the plastid genome of *T*. *tetragonolobum* is the second largest among the Lamiales species studied; only 2.1 kbp smaller than that of *O*. *europaea*, and approximately 0.2–0.5 kbp larger than those of *B*. *hygrometrica* or *S*. *indicum* (Table 1). Length variation in specific regions of the chloroplast genome was also observed, with *T*. *tetragonolobum* having the smallest LSC and SSC regions, but the largest IR region (Table 1) among the Lamiales cp genomes analyzed.

**Fig 3:**
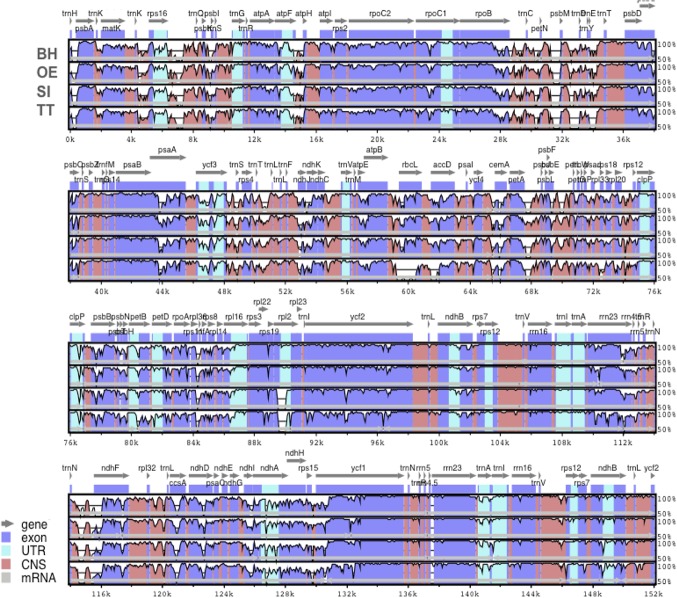
Percent identity plot comparing the chloroplast genomes of four species of Lamiales, using *Nicotiana tabacum* as reference. Vertical scale indicates the percentage of identity, ranging from 50% to 100%. Horizontal axis indicates the coordinates within the chloroplast genome. Arrows indicate the annotated genes and their transcriptional direction. Genome regions are color coded as exon, untranslated region (UTR), conserved noncoding sequences (CNS), and mRNA. BH: *Boea hygrometrica*; OE: *Olea europaea*; SI: *Sesamum indicum*, and TT: *Tanaecium tetragonolobum*.

Sequence identity comparisons between the four Lamiales chloroplast genomes were performed using the software mVISTA [41] with the annotation of *Nicotiana tabacum* as reference (Fig 3). The complete aligned sequences indicate that Lamiales chloroplast genomes are rather conservative, although some divergent regions are also found. As seen in other flowering plants [7,8,50], coding regions were more conserved than their noncoding counterparts. Our analysis showed that the most divergent coding regions in the four Lamiales chloroplast genomes analyzed were *ycf1*, *ycf2*, *ndhF*, *rbcL*, *accD*, *psaA* and *rpl2* (Fig 3). Indeed, the *ycf1* and *accD* coding regions have also been observed as divergent regions in plastid genomes of other angiosperms [7,8,11,50], representing good markers for phylogenetic studies. Noncoding regions showed higher sequence divergence among Lamiales chloroplast genomes, with the *trnH-GUG/psbA*, *psbM/trnD-GUC*, *petA-psbJ*, and *rps16-trnQ-UUG* regions having the highest levels of divergence (Fig 3). Some of these chloroplast noncoding regions have also been used in phylogenetic studies [50,52,53].

Inverted repeat (IR) expansion and contraction are common evolutionary events in plants [6–11]. In fact, the locations of the LSC/IR and SSC/IR junctions are sometimes regarded as an index of chloroplast genome evolution [10]. To evaluate the potential impact of these changes in the chloroplast genome of *T*. *tetragonolobum*, we compared the boundaries of IR regions with those from other Lamiales species (Fig 2). In all four Lamiales chloroplast genomes analyzed, the boundary between the LSC and IR regions was located within the *rps19* gene, resulting in the formation of an *rps19* pseudogene. The largest length of *rps19* pseudogene in the Lamiales (100 bp) was observed in *Boea hygrometrica* (Fig 2). The boundary of the SSC/IR junction in Lamiales chloroplast genomes was located within the *ycf1* gene, also resulting in the formation of a *ycf1* pseudogene, which varied in length between 816 bp and 1,301 bp (Fig 2). As observed in other chloroplast genome studies [6–9], the IR expansion/contraction in Lamiales has led to changes in the structure of the chloroplast genome, contributing to the formation of pseudogenes.

Our analysis also indicated that genome size variation between species of Lamiales might be mostly due to length differences in noncoding regions (intergenic spacers and introns), as well as to gene losses or gains (Table 1). Nevertheless, the gene content between species of Lamiales is very similar. Zhang and co-workers [10] reported nine small genes of unknown function for *Boea hygrometrica* (*ccs1*, *ycf10*, *ycf33*, *ycf37*, *ycf41*, *ycf54*, *ycf89*, *orf93*, and *trnL-GAG*) and indicated that those genes are not presented in *O*. *europaea* [49] or *Sesamum indicum* [10]. Despite that, a closer look into the original sequences generated in those studies indicated that all nine genes were also present in those two species. Gene losses were observed in all four Lamiales chloroplast genomes analyzed. One tRNA gene (*trnS-CGA*) and one protein-coding gene (*psbG*) were only found in the *Olea europaea* genome. On the other hand, the protein-coding gene *ycf68* was not observed in the *O*. *europaea* chloroplast genome. Unlike other Lamiales, some introns are lacking in the plastome of *T*. *tetragonolobum* (*trnG-UCC*, *rps12*, *petB*, *petD*, *rpl16*, and *rps19*), although the coding sequences of the genes that contain those introns remain intact.

### Comparison of the evolutionary rates of protein-coding sequences

A comparison of base substitutions in the chloroplast genomes of *Boea hygrometrica*, *Olea europaea*, *Sesamum indicum*, and *Tanaecium tetragonolobum* was conduced and the estimated values for each gene are provided as supplemental data (S1 Table). Our results showed that evolutionary rates of chloroplast genes are not uniform. Evolutionary rate heterogeneity was also reported for other plant species [10,54–57]. Although the causes and consequences of the differences of evolutionary rates among encoding genes remain under debate, some studies have reported that such differences can be attributed to generation time, relaxed selection, length of the encoded products, gene expression level, and gene function [55,58–60]. In fact, for all species of Lamiales analyzed, some genes involved in photosynthesis function such as *atpH*, *psbM*, *psbF*, *petG*, *psaJ*, and *psbT* evolved slower and presented values of Ka/Ks equal to 0.001 (S1 Table). In contrast, other genes such as the protein-coding sequences of the small subunit of ribossome *rps7*, *rps12*, and *rps12_3end*, and genes with unclear functions such as *ycf2* and *ycf15*, evolved faster with values of Ka/Ks higher than 0.5 (S1 Table). In addition, the comparisons of evolutionary rates of 84 chloroplast genes between the four species of Lamiales analyzed showed that eight genes (*psbk*, *rpoC1*, *rpl33*, *rps12*, *rpoA*, *rpl14*, *rpl2*, and *ycf2*) in the *T*. *tetragonolobum* cp genome evolved rapidly (S1 Table). However, some protein-coding sequences with slow evolutionary rates were also observed in *T*. *tetragonolobum*, including *matK*, *atpF*, *ihbA*, and *psbL*.

The weighted average of substitutions rates for all chloroplast regions (i.e., LSC, IR, and SSC) of the four studied taxa recovered similar Ka/Ks ratios between IR regions (S2 Table), with values ranging from 0.570 (*T*. *tetragonolobum*) to 0.621 (*O*. *europaea*). Although the weighted average values of Ka and Ks were higher in the SSC region for all species of Lamiales, the weighted average values of Ka/Ks ratios were higher in the IR region (S2 Table). In contrast to the non-synonymous substitution rates, synonymous substitution rates changed proportionally across genes of all Lamiales species studied (with exception of *ycf1*, *rpl22*, *psaJ*, and *matK*). These results are in agreement with earlier findings by Muse and Gaudt [55].

Overall, our results indicate that the evolutionary rates of genes in Lamiales are lineage-, locus-, and region-specific, further corroborating the observation that the evolutionary pattern of nucleotide substitution in chloroplast genomes of flowering plants is complex [10,54,55,61,57].

### Repeat sequence analyses

In population genetic studies of angiosperms, coupling biparentally inherited nuclear markers with those derived from chloroplast genomes generally associated with maternal inheritance (but see [62]), allows us to better understand the contributions of seed and pollen dispersal events to population processes associated with plant evolution [63,64]. Nuclear and chloroplast microsatellite molecular markers (nSSRs and cpSSRs, respectively) can be easily identified in whole genome sequences by *in silico* searches [47,65,66]. These markers have been developed or cross-amplified in a plethora of taxa [65,67–71]. Through *in silico* analyses of occurrence, type, and distribution of cpSSRs in the *T*. *tetragonolobum* plastome, we identified a total of 347 cpSSRs (Table 3). Among those, mono- and trinucleotide repeats were the most common, representing 74.9% (260 cpSSRs) and 18.7% (54 cpSSRs) of all nucleotide repeats identified in the present study (Table 3). No pentanucleotide tandem repeat was identified and low frequencies of di-, tetra-, and hexanucleotide repeats were observed in the *T*. *tetragonolobum* chloroplast genome (Tables 3 and 4). Among the 260 mononucleotide repeats, only 12 C/G type repeats were found, with all other repeats belonging to the A/T type. Repeat number of mononucleotide motifs ranged from seven (52.7%) to 13. On the other hand, *in silico* searches for repetitive elements in *Olea europaea* identified 305 repetitive sequences, 96% of which were mononucleotide SSRs with seven or more repeat units [49]. For *T*. *tetragonolobum*, we observed a plethora of SSRs, many of which are mononucleotide repeats in noncoding regions of the chloroplast genome. For instance, 182 (70%) mononucleotide repeats were identified in noncoding regions, including 172 in intergenic regions and ten in introns. The number of mononucleotide tandem repeats found in noncoding regions of the *T*. *tetragonolobum* plastid genome was much greater than those recorded for other species of flowering plants [8,65,66]. Tandem repeats located in the noncoding regions of the plastid genome generally show intraspecific variation in repeat number [72,73]. Therefore, noncoding regions of the chloroplast genome that are currently being used for phylogenetic studies in angiosperms [52] might also represent good regions for the development of polymorphic cpSSRs molecular markers.

**Table 3:** Total number of perfect simple sequence repeats (SSRs) identified within the chloroplast genome of *Tanaecium tetragonolobum* (Jacq.) L.G. Lohmann.

SSR sequence	Number of repeats											Total
	3	4	5	6	7	8	9	10	11	12	13	
A/T	-	-	-	-	128	56	33	18	6	3	4	248
C/G	-	-	-	-	9	1	1	1	-	-	-	12
AC/CA	-	3	-	-	-	-	-	-	-	-	-	3
TA/AT	-	6	4	1	1	-	-	-	-	-	-	12
TC/CT	-	3	-	-	-	-	-	-	-	-	-	3
GA/AG	-	6	-	-	-	-	-	-	-	-	-	6
GT/TG	-	2	-	-	-	-	-	-	-	-	-	2
CAN/CTN/CCT	8	1	-	-	-	-	-	-	-	-	-	9
GAN/GCA/GTA	4	-	-	-	-	-	-	-	-	-	-	4
AAN/ATN/AGN	16	2	-	-	-	-	-	-	-	-	-	18
TAN/TTN/TCN/TGN	20	3	-	-	-	-	-	-	-	-	-	23
GTCT/TAAA/CTTT	3	-	-	-	-	-	-	-	-	-	-	3
GAAA/TCTT/AATC	3	-	-	-	-	-	-	-	-	-	-	3
ATTAGT	-	-	-	1	-	-	-	-	-	-	-	1
Total												347

N indicates a degenerate base (not a gap) following the IUPAC system.

**Table 4:** Distribution of tetra- and hexanucleotide simple sequence repeats (SSRs) in the chloroplast genome of *Tanaecium tetragonolobum* (Jacq.) L.G.

SSR type	SSR sequence	Size	Start	End	Location
tetranucleotide	GTCT	12	11539	11550	*atpA* (CDS)
tetranucleotide	TAAA	12	42570	42581	*psaA/ycf3* (IGS)
tetranucleotide	CTTT	12	48538	48549	*trnF-GAA/ndhJ* (IGS)
tetranucleotide	GAAA	12	61253	61264	*ycf4/cemA* (IGS)
tetranucleotide	TCTT	12	72029	72040	*rps12/psi_psbT* (IGS)
tetranucleotide	AATC	12	123975	123986	*rps15/ycf1* (IGS)
hexanucleotide	ATTAGT	36	55654	55688	*atpB/rbcL* (IGS)

Lohmann. CDS = coding sequence, IGS = intergenic spacers.

We identified 20 cpSSRs markers distributed in noncoding regions of the *T*. *tetragonolobum* chloroplast genome (S3 Table). Given that flanking regions of SSRs are highly conserved across taxa [74,75], we also searched for inter-specific SSR variation in this set of cpSSRs in other three species of Lamiales (*B*. *hygrometrica*, *O*. *europaea*, and *S*. *indicum*). However, primer similarity declines with evolutionary distance between focal species [76,77], and we were only able to identify SSR variation between *T*. *tetragonolobum* and *Sesamum indicum* (Pedaliaceae) in four primer pairs (S3 Table). We expect the potential set of SSR markers identified in the noncoding regions to be easily amplified and variable between individuals and populations of *T*. *tetragonolobum*. However, the characterization of these cpSSRs markers was beyond the scope of this project.

Apart from SSRs, dispersed repeats are also thought to play an important role in genome recombination and rearrangement [78]. In the plastid genome of *T*. *tetragonolobum*, we found 28 (forward) repeats and 19 inverted (palindrome) repeats of at least 30 bp per repeat-unit with a sequence identity of more than 90% (Table 5); these repeats were mostly found in noncoding regions (61.7%), with the three largest repeats including 64 bp. The repeat structure of other three Lamiales species was also analyzed using REPuter. The number of repeat sequences in *T*. *tetragonolobum* was higher than that of *S*. *indicum* which has 15 repeats (seven forward and eight inverted), *B*. *hygrometrica* which has eight repeats (five forward and three inverted), and *O*. *europaea* which has three repeats (one forward and two inverted). Of the four Lamiales plastid genomes analyzed, *T*. *tetragonolobum* contains the greatest total number of repeats with 40 bp or longer. Variation in the number of repeat sequences has been observed between species belonging to different families and even between co-generic species [8]. The dispersed repeats identified in *T*. *tetragonolobum* provide a basis for the development of markers for phylogenetic and population genetic studies.

**Table 5:** Sequence repeats in the chloroplast genome of *Tanaecium tetragonolobum* (Jacq.) L.G. Lohmann.

Repeat no.	Repeat size (bp)	Repeat start 1	Repeat start 2	Type	Location of repeat 1	Location of repeat 2
1	32	147046	147064	F	*ycf2*	*ycf2*
2	32	129711	129808	F	*ycf1/trnN-GUU* *	*ycf1/trnN-GUU* *
3	32	109335	129808	P	*trnN-GUU/ycf1* *	*ycf1/trnN-GUU* *
4	32	109238	129711	P	*trnN-GUU/ycf1* *	*ycf1/trnN-GUU* *
5	32	109238	109335	F	*trnN-GUU/ycf1* *	*trnN-GUU/ycf1* *
6	32	91876	147064	P	*ycf2*	*ycf2*
7	32	91858	147046	P	*ycf2*	*ycf2*
8	32	91858	91876	F	*ycf2*	*ycf2*
9	33	145723	145744	F	*ycf2*	*ycf2*
10	33	93198	145744	P	*ycf2*	*ycf2*
11	33	93177	145723	P	*ycf2*	*ycf2*
12	33	93177	93198	F	*ycf2*	*ycf2*
13	35	58186	58315	F	*rbcL/accD* *	*rbcL/accD* *
14	35	58109	58238	F	*rbcL/accD* *	*rbcL/accD* *
15	35	57361	57418	F	*rbcL/accD* *	*rbcL/accD* *
16	36	58149	58278	F	*rbcL/accD* *	*rbcL/accD* *
17	37	129772	129790	F	*ycf1/trnH-GUU* *	*ycf1/trnH-GUU* *
18	37	109269	129790	P	*trnH-GUU/ycf1* *	*ycf1/trnH-GUU* *
19	37	109251	129772	P	*trnH-GUU/ycf1* *	*ycf1/trnH-GUU* *
20	37	109251	109269	F	*trnH-GUU/ycf1* *	*trnH-GUU/ycf1* *
21	39	60281	60302	F	*psaI/ycf4* *	*psaI/ycf4* *
22	40	147005	147023	F	*ycf2*	*ycf2*
23	40	91909	147023	P	*ycf2*	*ycf2*
24	40	91891	147005	P	*ycf2*	*ycf2*
25	40	91891	91909	F	*ycf2*	*ycf2*
26	40	58855	58900	F	*rbcL/accD* *	*rbcL/accD* *
27	41	120660	140006	P	*ndhA* (intron)	*trnV-GAC/rps12_3end* *
28	41	98907	120660	F	*trnV-GAC/rps12_3end* *	*ndhA* (intron)
29	43	151949	151991	F	*trnI-CAU/rpl23* *	*trnI-CAU/rpl23* *
30	43	86962	151991	P	*rpl23/trnI-CAU* *	*trnI-CAU/rpl23* *
31	43	86920	151949	P	*rpl23/trnI-CAU* *	*trnI-CAU/rpl23* *
32	43	86920	86962	F	*rpl23/trnI-CAU* *	*rpl23/trnI-CAU* *
33	44	149605	149632	F	*ycf2*	*ycf2*
34	44	89305	149632	P	*ycf2*	*ycf2*
35	44	89278	149605	P	*ycf2*	*ycf2*
36	44	89278	89305	F	*ycf2*	*ycf2*
37	44	68866	68911	F	*rps18*	*rps18*
38	44	58810	58900	F	*rbcL/accD* *	*rbcL/accD* *
39	49	135522	136455	F	*trnI-GAU/ycf68* *	*trnI-GAU/rrn16* *
40	49	125188	125212	F	*ycf1*	*ycf1*
41	49	103507	136455	P	*trnI-GAU/ycf68* *	*ycf68/trnI-GAU* *
42	50	102450	135521	P	*rrn16/trnI-GAU* *	*trnI-GAU/ycf68* *
43	50	102450	103507	F	*rrn16/trnI-GAU* *	*ycf68/trnI-GAU* *
44	51	58799	58844	F	*rbcL/accD* *	*rbcL/accD* *
45	64	151949	151970	F	*trnI-CAU/rpl23* *	*trnI-CAU/rpl23* *
46	64	86941	151970	P	*rpl23/trnI-CAU* *	*trnI-CAU/rpl23* *
47	64	86920	151949	P	*rpl23/trnI-CAU* *	*trnI-CAU/rpl23* *

Type are F (forward) and P (palindrome) repeats

* intergenic spacers.

## Conclusions

In this study, we assembled and analyzed the complete nucleotide sequence of the chloroplast genome of *T*. *tetragonolobum*, the first fully sequenced plastome in the Bignoniaceae. This plastome was compared to three other plastomes of representatives of the Lamiales providing interesting insights on the evolution of the chloroplast genomes within this important angiosperm order. No significant structural changes were found among the chloroplast genomes of the Lamiales taxa analyzed (i.e., *B*. *hygrometrica*, *O*. *europaea*, *S*. *indicum*, and *T*. *tetragonolobum*). However, the chloroplast genomes of the four Lamiales species showed variation in size due to the expansion or contraction of the IR region as well as variation in the length of intergenic spacers. The discovery of tandem repeats within the chloroplast genome of *T*. *tetragonolobum* and the presence of divergent regions between chloroplast genomes within Lamiales provides useful information for future phylogenetic, phylogeographic and evolutionary studies in this order.

## Supporting Information

S1 Table Comparisons of the evolutionary rates of 84 genes between the chloroplast genomes of four Lamiales plant species: *Boea hygrometrica* (Bunge) R. Br., *Olea europaea* L., *Sesamum indicum* L., and *Tanaecium tetragonolobum* (Jacq.) L.G. Lohmann. (XLSX)

S2 Table Weighted average of evolutionary rates across chloroplast genome structures (LSC: large single copy, IR: inverted repeat, and SSC: small single copy) for four Lamiales plant species: *Boea hygrometrica* (Bunge) R. Br., *Olea europaea* L., *Sesamum indicum* L., and *Tanaecium tetragonolobum* (Jacq.) L.G. Lohmann. (DOCX)

S3 Table Set of 20 microsatellite loci distributed in noncoding regions and designed for *Tanaecium tetragonolobum* (Jacq.) L.G. Lohmann, followed by locus name, primer sequence (F: forward and R: reverse), repeat motif, and expected fragment size. Underlined locus names represent SSR loci shared with *Sesamum indicum*.
(DOCX)
