# Supplementary material for: Complete Chloroplast Genome of Tanaecium tetragonolobum: The First Bignoniaceae Plastome
Source: PLoS One. 2015 Jun 23;10(6):e0129930. doi: 10.1371/journal.pone.0129930 (PMC4478014; doi:10.1371/journal.pone.0129930)
Supplement: S2 Table — (DOCX) [file pone.0129930.s002.docx]

**Supporting Information Table S2**. Weighted average of evolutionary rates across chloroplast genome regions (LSC: Large Single Copy, IR: Inverted Repeat, and SSC: Small Single Copy) of four species of Lamiales: *Boea hygrometrica* (Bunge) R. Br., *Olea europaea* L., *Sesamum indicum* L., and *Tanaecium tetragonolobum* (Jacq.) L.G. Lohmann.

| cpDNA structure | **Species** | ***Ka*** | ***Ks*** | ***Ka/Ks*** |
| --- | --- | --- | --- | --- |
| *LSC* | *B. hygrometrica* | 0.0394 | 0.3070 | 0.1284 |
|  | *O. europaea* | 0.0247 | 0.2107 | 0.1202 |
|  | *S. indicum* | 0.0284 | 0.2309 | 0.1259 |
|  | *T. tetragonolobum* | 0.0631 | 0.2414 | 0.2357 |
| *IR* | *B. hygrometrica* | 0.0234 | 0.3879 | 0.6029 |
|  | *O. europaea* | 0.0128 | 0.0297 | 0.5711 |
|  | *S. indicum* | 0.0199 | 0.0376 | 0.6219 |
|  | *T. tetragonolobum* | 0.0348 | 0.0588 | 0.5701 |
| *SSC* | *B. hygrometrica* | 0.1096 | 0.4601 | 0.2382 |
|  | *O. europaea* | 0.0847 | 0.3412 | 0.2362 |
|  | *S. indicum* | 0.0861 | 0.3596 | 0.2386 |
|  | *T. tetragonolobum* | 0.0838 | 0.3549 | 0.2361 |
