# Supplementary material for: Complete Chloroplast Genome of Tanaecium tetragonolobum: The First Bignoniaceae Plastome
Source: PLoS One. 2015 Jun 23;10(6):e0129930. doi: 10.1371/journal.pone.0129930 (PMC4478014; doi:10.1371/journal.pone.0129930)
Supplement: S3 Table — Underlined locus names represent SSR loci shared with Sesamum indicum. (DOCX) [file pone.0129930.s003.docx]

**Supporting Information Table S3.** Set of 20 microsatellite loci distributed in noncoding regions and designed for *Tanaecium tetragonolobum* (Jacq.) L.G. Lohmann, including locus name, primer sequence (F: forward and R: reverse), repeat motif, and expected fragment size. Underlined locus names represent SSR loci shared with *Sesamum indicum*.

| SSR | Sequence (5’-3’) | Repeat motif | Size (bp) |
| --- | --- | --- | --- |
| *Tan01* | F: ACCTGAGCCACAGAAGATGC | (GTCT)_3_ | 189 |
|  | R: TCTTCAAACCGGGCTTATTG |  |  |
| *Tan02* | F: CCCCTGTATGGCTTCTTCAA | (T)_13_ | 172 |
|  | R: TCCAAGAGAAACTCCCATCG |  |  |
| *Tan03* | F: GCCTTTCCACTTCGATCAAT | (A)_10_ | 196 |
|  | R: CATCGAAGAAATCCCAAACC |  |  |
| *Tan04* | F: GGGCGTAGCTACCGAGATTA | (TTC)_4_ | 191 |
|  | R: GAGGGGTCATGGAAAGAACA |  |  |
| *Tan05* | F: GGGTCCAACTTCTTGGATGA | (TA)_5_ | 223 |
|  | R: TCGAACCCACATCTTCTCCT |  |  |
| *Tan06* | F: CTCTTTCCACAAAGCGAAGG | (TA)_7_ | 177 |
|  | R: CGAGGCGTTTCGAATAAAAG |  |  |
| *Tan07* | F: GGGAAAATGATCCCACAAAG | (A)_11_ | 191 |
|  | R: CGGAATAGTTCCCTTCGTTC |  |  |
| *Tan08* | F: TGCGATGCTCTAACCTCTGA | (ATA)_4_ | 202 |
|  | R: TCGCTACATAAATCGTTCTGTTCT |  |  |
| *Tan09* | F: GCCAGTTCAAGTCTGGTTCC | (CTTT)_4_ | 150 |
|  | R: ATGGGGTATATCTCGCCAAG |  |  |
| *Tan10* | F: CCCCCTTGTCCCTTTAATTG | (TAA)_4_ | 156 |
|  | R: CATGGACCAGGAACCAGAC |  |  |
| *Tan11* | F: AGGGTTGACATAGGGGTTGA | (ATTAGT)_6_ | 222 |
|  | R: TCGACACGAATGAATTAGGC |  |  |
| *Tan12* | F: CCACATCCCTATTTCCTCCA | (TA)_6_ | 176 |
|  | R: CCCCTCTTAGCGAGTAGTTCC |  |  |
| *Tan13* | F: TCGTCGCGCGATATATAAAA | (A)_15_ | 240 |
|  | R: TGAAAAGTGAAGAAATTTCAAG |  |  |
| *Tan14* | F: GCGAACAACTCTTTTTCTAGCC | (T)_12_ | 240 |
|  | R: TCGTGATTGGGGTATTTGAA |  |  |
| *Tan15* | F: AATTCCCGAATTCCATTTCC | (A)_10_ | 205 |
|  | R: GGGGAAATAAGACTCCTCAGC |  |  |
| *Tan16* | F: CCATAGAACATCTGGCGTAACA | (AATA)_3_ | 156 |
|  | R: GAGTGGTTTTGTTGCCGAAT |  |  |
| *Tan17* | F: AACCGAAATGAATGAATTTTTG | (AATC)_3_ | 225 |
|  | R: TCGATGCGAAAATGTACCAA |  |  |
| *Tan18* | F: CTTTCAACCGAGGCTAGAAGAA | (T)_13_ | 250 |
|  | R: CCGTTCTCCCACAACAAAAA |  |  |
| *Tan19* | F: CAATCGAGTCCTTGTTTCGA | (T)_12_ | 179 |
|  | R: GAAGGATATCCCCGCATAC |  |  |
| *Tan20* | F: TCCAAATCCATGCAACTCAA | (GAAA)_3_...(T)_12_ | 192 |
|  | R: GGTCGATTGACAAAAAGGAAA |  |  |
